# Supplementary material for: Identification of photoperiod-induced specific miRNAs in the adrenal glands of Sunite sheep (Ovis aries)
Source: Front Vet Sci. 2022 Jul 22;9:888207. doi: 10.3389/fvets.2022.888207 (PMC9354845; doi:10.3389/fvets.2022.888207)
Supplement: Supplementary file 1 [file Data_Sheet_1.ZIP › Supplementary file/miRDeep2 file.zip!/miRDeep2 file/Know_Structure/LP42A1/oar-mir-376c.pdf]

miRBase precursor : oar-mir-376c
Total read count : 698
oar-miR-376c-5p read count : 242
oar-miR-376c-3p read count : 455
remaining reads : 1

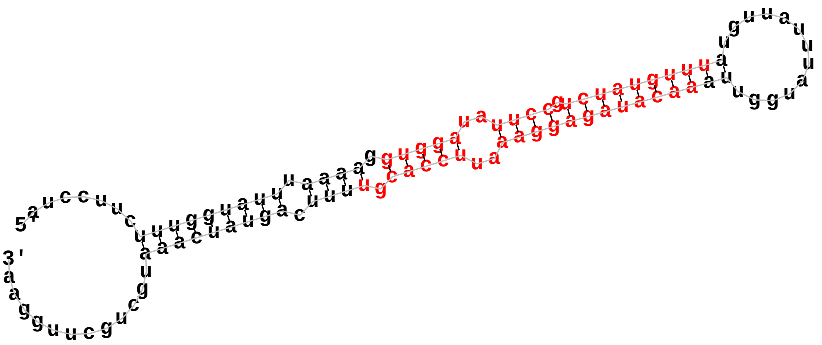

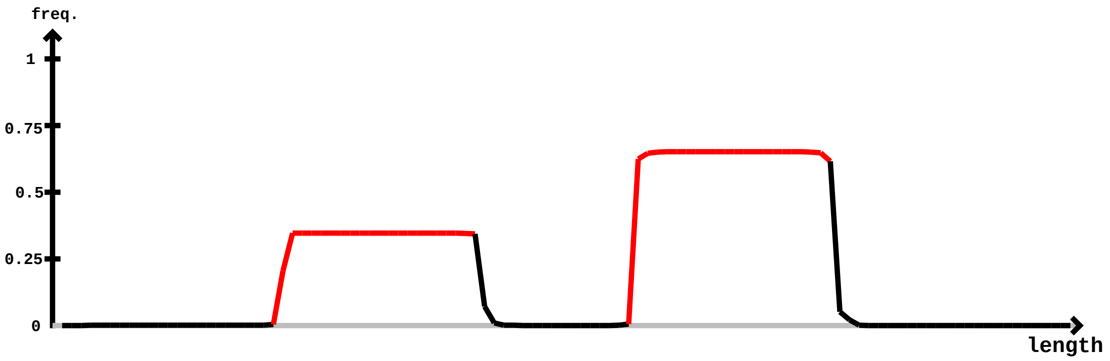


```
                                                                   oar-miR-376c-3p
                              oar-miR-376c-5p
5'- auccuucuuugguauuuaaaagguggauauuccgucuauguuuauguuauuuaugguuaaacauagaggaaauuccacguuucaguaucaaaugcugcuuggaa   -3'    exp
    .......((((((((.((((.(((((..((((.((((((((((............))))))))))))))..))))).)))).))))))))..............    reads    mm    sample
    ..ccuucuuugguauuuaaaag..................................................................................    1        0     seq
    .....................gguggauauuccgucuauguuu.............................................................    2        0     seq
    ......................guggauauuccgucuaugu...............................................................    1        0     seq
    ......................guggauauuccgucuauguu..............................................................    1        0     seq
    ......................guggauauuccgucuauguuu.............................................................    113      0     seq
    ......................guggauauuccgucuauguuua............................................................    26       0     seq
    .......................uggauauuccgucuauguuu.............................................................    75       0     seq
    .......................uggauauuccgucuauguuua............................................................    17       0     seq
    .......................uggauauuccgucuauguuuau...........................................................    6        0     seq
    .......................uggauauuccgucuauguuuaugu.........................................................    1        0     seq
    .........................................................uaaacauagaggaaauuccacg.........................    1        0     seq
    ..........................................................aaacauagaggaaauuccacgu........................    3        0     seq
    ...........................................................aacauagaggaaauucca...........................    1        0     seq
    ...........................................................aacauagaggaaauuccac..........................    2        0     seq
    ...........................................................aacauagaggaaauuccacg.........................    21       0     seq
    ...........................................................aacauagaggaaauuccacgu........................    389      0     seq
    ...........................................................aacauagaggaaauuccacguu.......................    17       0     seq
    ...........................................................aacauagaggaaauuccacguuu......................    2        0     seq
    ............................................................acauagaggaaauuccacgu........................    2        0     seq
    ............................................................acauagaggaaauuccacguu.......................    4        0     seq
    ............................................................acauagaggaaauuccacguuu......................    9        0     seq
    .............................................................cauagaggaaauuccacguuu......................    3        0     seq
    ..............................................................auagaggaaauuccacguuuu.....................    1        0     seq
```
